# Cost-Aware Online Switching of Four-Qubit Probe States for Quantum Magnetometry Under Non-Stationary Noise

**DOI:** 10.3390/s26206428

**Published:** 2026-10-11

**Authors:** Diwakar Diwakar, Varsha Santosh Sambhaje

**Affiliations:** Quantum Technology Institute & Department of Computer Science and Engineering, SRM University-AP, Amaravati 522240, Andhra Pradesh, India

**Keywords:** quantum magnetometry, quantum sensing, online learning, non-stationary noise, GHZ states, adaptive control, finite-shot simulation

## Abstract

Quantum magnetometry can be enhanced through the use of entangled probe states, yet the optimal probe selection depends greatly on the noise, readout errors, and costs involved in preparation. An analysis is given of a simulation of a four-qubit magnetic field estimation experiment where the optimal probe state is adaptively chosen via an online controller that changes between product and GHZ probes using delayed and self-consistent feedback without knowing the underlying noise model. The performance of the controller is compared to that of a validation-selected fixed probe state strategy, a hindsight fixed probe strategy, a fixed product probe strategy, a fixed GHZ probe strategy, a fixed graph-state probe strategy, and an oracle-based dynamic probe strategy in eight stationary and non-stationary noise environments. In the actual experiment, 20 random seeds, 1200 timesteps per noise scenario, and 2000 measurement shots per timestep in the case of local dephasing with readout error are used. The sliding-window controller yields an overall RMSE of 0.015542 versus 0.017305 for the validation-selected fixed probe state, 0.017330 for the fixed product, 0.017821 for fixed GHZ, and 0.061184 for the fixed graph state. A paired permutation test on 160 trial cases gives the mean RMSE difference of −0.001763 versus the validation-selected fixed probe state with $p=9.99\times 10^{-4}$. The adaptive algorithm improves performance in seven out of eight noise environments but still does not do well enough in the constant high-noise scenario at 5.4% worse than the global fixed baseline in the constant-high-noise scenario.

## 1. Introduction

Quantum sensing seeks to measure physical quantities with enhanced precision due to the benefits of quantum coherence, squeezing, and entanglement [1,2,3,4]. Sensing of the magnetic field is a primary example since one may exploit the field-dependent phase shift acquired by a carefully prepared quantum sensor and recovered from its measurement outcomes. While for the case of the ideal noiseless environment, maximally entangled states have an advantageous scaling for the phase estimation task, in realistic cases of finite number of runs and noisy environments [5,6,7]. The conflict between enhanced sensitivity by entanglement and noise resistance is an incentive to select probes adaptively. Choosing a probe globally for the entire process can be inefficient if the surrounding conditions of the measurement are switching between low noise regimes, jumps, spikes, drifting, and persistently high noise regimes. On the other hand, a switching control that switches excessively will waste efforts on preparing unnecessary probes. The problem can then be formulated similar to the non-stationary bandit problem [8,9,10,11].

**Figure 1:**
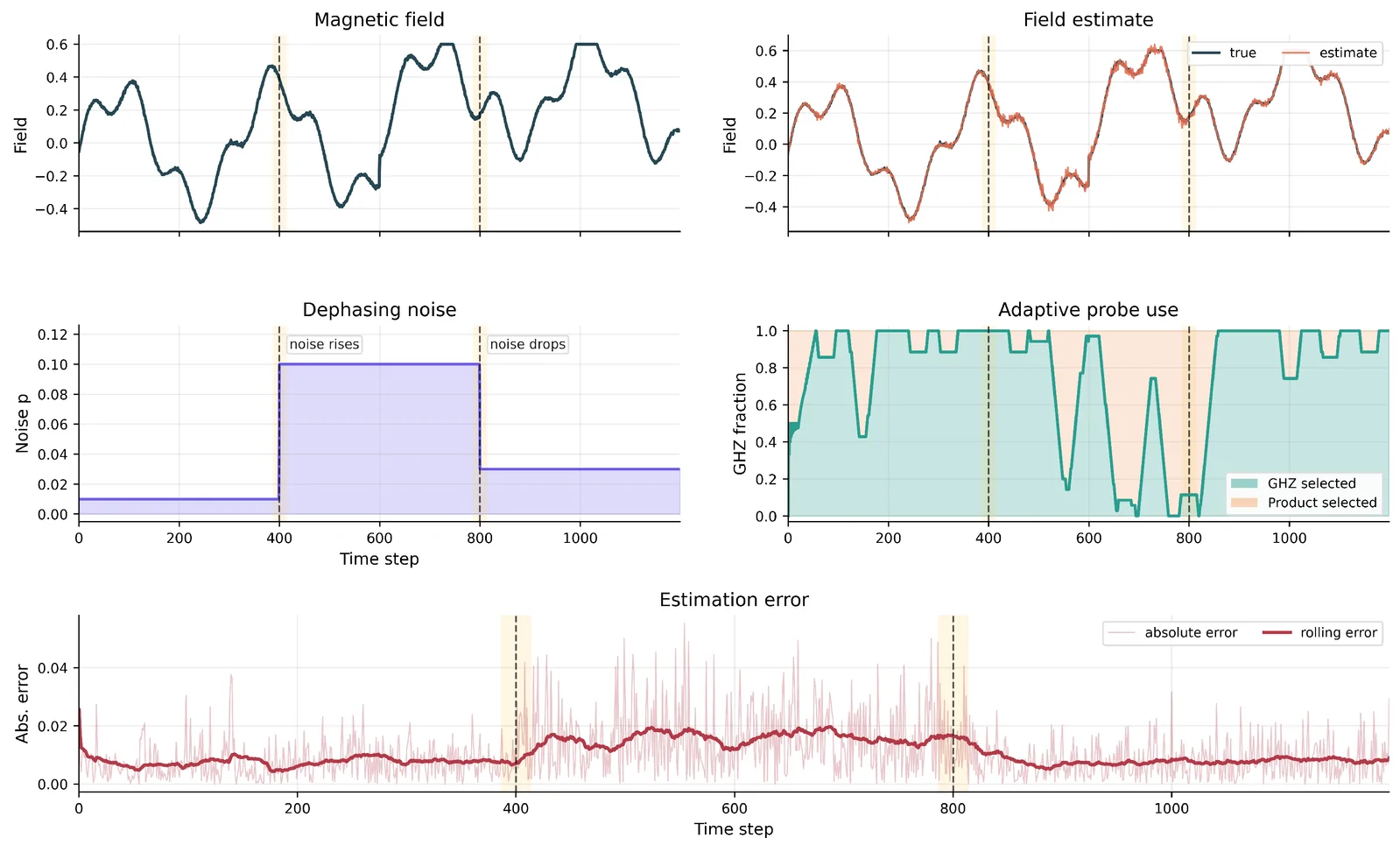
Input–output example for the sudden-change scenario.

This work considers a small four-qubit architecture that is created to be mechanically clear. The set of possible probes includes a product probe, a GHZ probe, and a linear graph-state probe. The suggested online control is based on the use of product and GHZ probes in order to make decisions, while the graph state is kept as an experimental probe for analysis of the effects of ablation. The control evaluates the recent squared error of each probe within a sliding window, performs pair checks to avoid a permanent fixation on one of the probes, and penalizes preparation and transition costs. The control does not get any direct noise labels. The behavior of the control under the sudden-change situation is illustrated in Figure 1.

The main contributions are as follows.

- A density-matrix simulation framework for four-qubit magnetometry using both stationary and non-stationary dephasing with readout noise.
- A resource-constrained sliding window controller with probe switching control based on delayed self-consistency and double check probing, without oracle noise.
- An experiment design to compare against static, validation chosen, hindsight, graph state, and dynamic oracle baseline methods on 160 seed scenarios.
- A writeup demonstrating aggregate RMSE gains that are statistically significant and a limitation in dealing with high levels of noise persistently.

## 2. Literature Review

Quantum estimation theory provides the statistical foundation for sensing protocols based on parameter-dependent quantum states [12,13,14,15]. In optical and interferometric settings, Caves showed that quantum noise can be reduced through squeezed-state resources [1]. Subsequent quantum-metrology work formalized the role of entanglement and collective measurements in beating standard quantum limits [2,3]. Reviews of quantum sensing and atomic-ensemble metrology summarize the broader landscape of achievable sensitivities, resource accounting, and experimental constraints [4,16]. Entangled probes are not universally superior under decoherence. The frequency-standard analysis of Huelga et al. established that maximally entangled probes can lose their advantage under independent dephasing [5]. Later work sharpened ultimate precision bounds in noisy metrology and showed how decoherence can erase Heisenberg-like scaling [6,7]. Related studies considered non-Markovian environments, dissipative bounds, noisy metrology beyond the standard quantum limit, and error-corrected sensing strategies [17,18,19,20,21,22]. These results indicate that the optimal probe is regime-dependent rather than universal. Further, magnetometry protocols often compare separable probes, GHZ-like probes, spin-squeezed states, and other entangled resources [23,24,25,26,27]. Graph and cluster states provide another route to structured multipartite entanglement [28,29,30]. Although graph states are valuable in measurement-based quantum computation and multipartite entanglement studies, their suitability for a specific sensing readout depends on the selected observable, calibration, and noise channel. Moreover, adaptive measurement has a separate history in phase estimation and optical sensing. Adaptive interferometry and homodyne schemes update measurement settings based on previous observations [31,32,33,34]. The present setting differs because the controller changes the probe state itself, operates with delayed proxy feedback, and must account for preparation and switching costs.

Online learning supplies concepts for balancing exploitation of the best current action with exploration under uncertainty. Multi-armed bandit theory gives finite-time regret tools for stationary and adversarial settings [8,9,35,36]. Switching bandits and non-stationary bandits add temporal drift and action-change costs [10,11]. Reinforcement-learning texts provide a broader view of sequential decision problems with delayed consequences [37]. The controller evaluated here is intentionally simpler than deep or black-box approaches [38]; interpretability and reproducible probe-risk accounting are prioritized.

## 3. Methodology

### 3.1. Data Description

These are simulated data obtained from the four-qubit density-matrix simulator. There are no hardware experimental data employed here. In each trial, there is a magnetic field time-series $B_{t}$, noise $p_{t}$, probe states, measurement counts, estimates ${\hat{B}}_{t}$ and controller metrics. The last experiment employs 20 seeds, eight scenarios, and 1200 time-steps per seed-scenario, resulting in 160 seed-scenario experiments and 192,000 controller time-steps data. Each time-step involves 2000 measurement shots. The scenarios examined include constant low, constant medium, constant high, sudden, gradual, alternating, burst, and random piecewise noise.

For the last run, dephasing using local error readout was applied. The value of the readout error probability was obtained as $p_{r}=0.0075$ for the resolved state. For the field model, the field range was mixed, and clipping to [−0.6, 0.6] was performed to avoid the phase ambiguity in the lightweight estimator.

### 3.2. Probe States and Field Encoding

The simulator uses exactly four qubits. The product probe is

(1) $$|\psi_{\mathrm{prod}}{\rangle =|+\rangle }^{⊗4}.$$

The GHZ probe is

(2) $$|\psi_{\mathrm{GHZ}}\rangle =\frac{|0000\rangle +|1111\rangle }{\sqrt{2}}.$$

The graph-state baseline is produced from ${|+\rangle }^{⊗4}$ by applying controlled-*Z* gates on edges (0, 1), (1, 2), and (2, 3). Field encoding is implemented by

(3) $$U\left(B_{t}\right)=\exp\left[-\frac{i}{2}\gamma \tau B_{t}\sum_{q=1}^{4}Z_{q}\right],$$

with γ=1 and interrogation time τ=1 in the final experiment.

Local dephasing is applied independently to each qubit. For a single qubit, the channel has Kraus representation

(4) $$D_{p}\left(\rho \right)=\left(1-p\right)\rho +pZ\rho Z.$$

The readout error model assumes that the measurement process is affected by independent bit flips. This is not an attempt at modeling all possible hardware errors but a simulation setup.

The quantity $B_{t}$ is a dimensionless field-type parameter. Given that the encoding employs $\phi =\gamma \tau B_{t}$ with γ=1 and τ=1, the values of RMSE are dimensionless phase-type errors. The physical magnetometer would have magnetic-field errors that scale as ${\mathrm{RMSE}}_{B}=\mathrm{RMSE}/\left(\gamma \tau \right)$. Hence, magnetic-field sensitivity will depend on the selected platform, gyromagnetic ratio, interrogation time, and measurement bandwidth.

The dephasing parameter $p_{t}$ is a dimensionless quantity. If we look at its Markovian interpretation, the dephasing channel employed locally has a coherence factor of $\left(1-2p_{t}\right)$ and is possibly connected to the dephasing time via $\left(1-2p_{t}\right)\approx \exp\left(-\tau /T_{2}\right)$. So, a higher value of $p_{t}$ implies shorter coherence time or increased dephasing due to the environment.

### 3.3. Probe Sensitivity and Dephasing Trade-Off

To understand the physics behind adaptive switching, we have applied the classical Fisher-information sensitivity analysis to the product and GHZ probes with the same dephasing and readout channel. Although the response of GHZ probe is more sensitive and acquires more phase compared to the product, the effect of local dephasing on its coherence is more pronounced. Further, Table 1 presents some values calculated from this analysis. In case of weak dephasing, the value of Fisher information in the GHZ state is much higher than that of the product probe. However, the advantage of the GHZ probe diminishes with the growth of dephasing, and the product probe starts being more convenient for p≈0.18 on evaluated grid. This provides a physical explanation for why the controller favors GHZ in low-noise regimes and increases product-probe use at higher noise.

### 3.4. Estimator

The estimator translates sampling results to estimates in the field using the calibrated response curve for each probe. The validation gate makes sure that the noiseless signed response has the right orientation and calibration prior to accepting any controller experiments. During the final run, all validations for the product, GHZ, and graph estimators succeed, achieving correlations and signs of 1.0. The biggest validation error in magnitude is less than $6\times 10^{-17}$.

### 3.5. Baselines

The following methods are evaluated:

- Fixed product: the product probe is used at every time step.
- Fixed GHZ: the GHZ probe is used at every time step.
- Fixed graph: the graph-state probe is used at every time step as an ablation baseline.
- Validation-selected fixed: a fixed probe is selected using validation performance rather than test-set performance.
- Sliding-window UCB: an adaptive non-stationary online-selection baseline that treats product and GHZ probes as two arms. It selects probes using recent delayed feedback and an exploration bonus over a sliding window but does not include the proposed controller’s paired-check mechanism, preparation-cost term, or explicit switching-cost term.
- Scenario-wise hindsight fixed: the best fixed probe is selected separately for each scenario after observing full scenario performance; this is an oracle-like retrospective baseline.
- Dynamic oracle: the best probe is selected at each time step using counterfactual squared errors; this is an unattainable lower-bound reference.

### 3.6. Proposed Method

For each probe a∈{product,GHZ}, the controller stores a recent history $H_{a}\left(t\right)$ of delayed squared-error feedback. The sliding-window risk estimate is

(5) $${\hat{R}}_{a,t}=\frac{1}{|H_{a}\left(t\right)|}\sum_{e\in H_{a}\left(t\right)}e,$$

where the final window size is 40. The controller selects the probe minimizing a cost-adjusted score

(6) $$S_{a,t}={\hat{R}}_{a,t}+\lambda c_{a}+\eta 1\left[a\neq a_{t-1}\right]+\alpha A_{a,t},$$

where $c_{a}$ is the preparation cost, λ=0.01, η=0.001, and $A_{a,t}$ is an age or uncertainty term controlled by the paired-check mechanism. Product and GHZ preparation costs are 0 and 3, respectively. The paired check is performed every 20 time steps and uses both probes to refresh risk estimates. A five-step feedback delay is applied. Table 2 summarizes the main steps of the proposed sliding-window probe-switching procedure.

In the main simulation benchmark, the delayed squared-error feedback is computed with respect to the known synthetic field trajectory. This should be interpreted as a controlled reference-feedback setting, not as unrestricted access to an unknown magnetic field during deployment. To test the practical sensitivity of the controller to this assumption, an additional noisy-reference feedback experiment was performed. In this variant, the controller update uses

(7) $$e_{a,t}^{\mathrm{ref}}={\left({\hat{B}}_{a,t}-B_{t}^{\mathrm{ref}}\right)}^{2},\;B_{t}^{\mathrm{ref}}=B_{t}+\epsilon_{t},\;\epsilon_{t}∼N\left(0,\;0.01^{2}\right).$$

This represents delayed feedback from a calibration signal, reference sensor, or controlled validation interval rather than exact ground-truth access.

**Figure 2:**
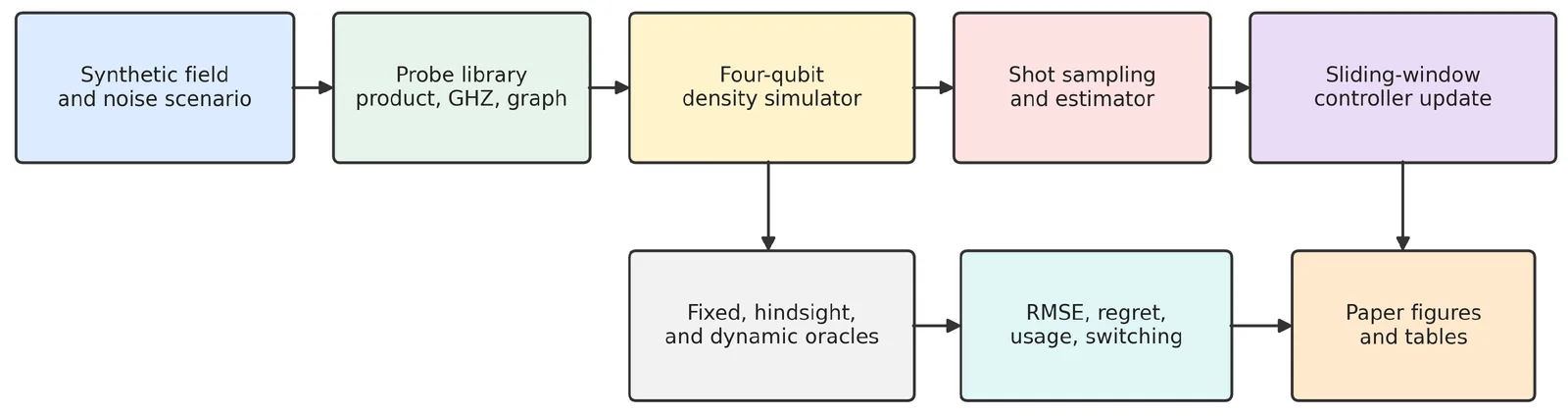
Workflow of the proposed adaptive probe-selection framework. Arrows indicate the sequential flow of information and control between processing stages, while colors are used only to visually distinguish the main components.

This evaluation process is illustrated in Figure 2. Simulated field and noise data are used to produce estimates specific to each probe by using the density matrix simulator for four qubits. These estimates are then utilized by the sliding window controller for selecting probes adaptively.

### 3.7. Experimental Setup

The final setup consists of 20 seeds, 1200 iterations, 2000 samples, action probes of product and GHZ, paired tests every 20 iterations, no cost for stale, window size of 40, feedback lag of 5, and switching cost weight of 0.001. Furthermore, a local hyperparameter sensitivity analysis was done based on five seeds and eight scenarios. The variables for sensitivity analysis were window size, switching cost weight, paired test interval, and feedback lag.

### 3.8. Evaluation Standards

The reported field values are dimensionless simulation variables. The field encoding uses ϕ=γτB, and the final experiments set γ=1 and τ=1. Therefore, the reported RMSE is an error in the normalized phase-like field variable. For a physical sensor with gyromagnetic ratio γ and interrogation time τ, the corresponding magnetic-field error would scale as ${\mathrm{RMSE}}_{B}=\mathrm{RMSE}/\left(\gamma \tau \right)$. The clipped interval [−0.6, 0.6] should be interpreted as the calibrated local dynamic range of the estimator, chosen to avoid phase ambiguity. The results therefore describe relative tracking performance within this local range, not an absolute hardware sensitivity or unlimited dynamic range.

As an illustrative physical mapping, consider an NV-center electronic-spin magnetometer with an electron-spin gyromagnetic ratio of approximately $\gamma_{e}=2\pi \times 28\;\mathrm{GHz}/T$. Using an illustrative interrogation time of τ=10μs, the aggregate normalized RMSE of the proposed controller, RMSE=0.015542, corresponds to an equivalent magnetic-field error of

(8) $${\mathrm{RMSE}}_{B}=\frac{0.015542}{\left(2\pi \times 28\times 10^{9}\right)\left(10\times 10^{-6}\right)}\approx 8.83\times 10^{-9}\;T=8.83\;\mathrm{nT}.$$

This conversion is provided only to illustrate the physical scale associated with the normalized simulation and should not be interpreted as an experimentally demonstrated magnetometer sensitivity. The achievable sensitivity of a practical NV-center magnetometer would additionally depend on the coherence time, spin initialization and readout fidelity, photon-collection efficiency, sensing bandwidth, pulse sequence, and other platform-specific noise and hardware characteristics. The primary error metric is root mean squared error,

(9) $$\mathrm{RMSE}=\sqrt{\frac{1}{T}\sum_{t=1}^{T}{\left({\hat{B}}_{t}-B_{t}\right)}^{2}}.$$

Additional metrics are mean absolute error,

(10) $$\mathrm{MAE}=\frac{1}{T}\sum_{t=1}^{T}\left|{\hat{B}}_{t}-B_{t}\right|,$$

bias,

(11) $$\mathrm{Bias}=\frac{1}{T}\sum_{t=1}^{T}\left({\hat{B}}_{t}-B_{t}\right),$$

and dynamic regret,

(12) $$G=\frac{1}{T}\sum_{t=1}^{T}\left[{\left({\hat{B}}_{a_{t},t}-B_{t}\right)}^{2}-\min_{a}{\left({\hat{B}}_{a,t}-B_{t}\right)}^{2}\right].$$

Significance of statistical analysis is based on seed-scenario differences for the proposed controller in relation to the selected fixed baseline through validation. Diagnostics of scenarios, which include agreement with oracle, probing, switch, and compliance to fixed-baseline tolerances of the proposed controller, are also captured in the acceptance report.

## 4. Results

### 4.1. Aggregate Performance

The aggregate results are presented in Table 3. The RMSEs of the proposed controller are lower than the RMSEs of the fixed, fixed product, fixed GHZ, and fixed graph controllers, which have been selected by the validation process.

**Figure 3:**
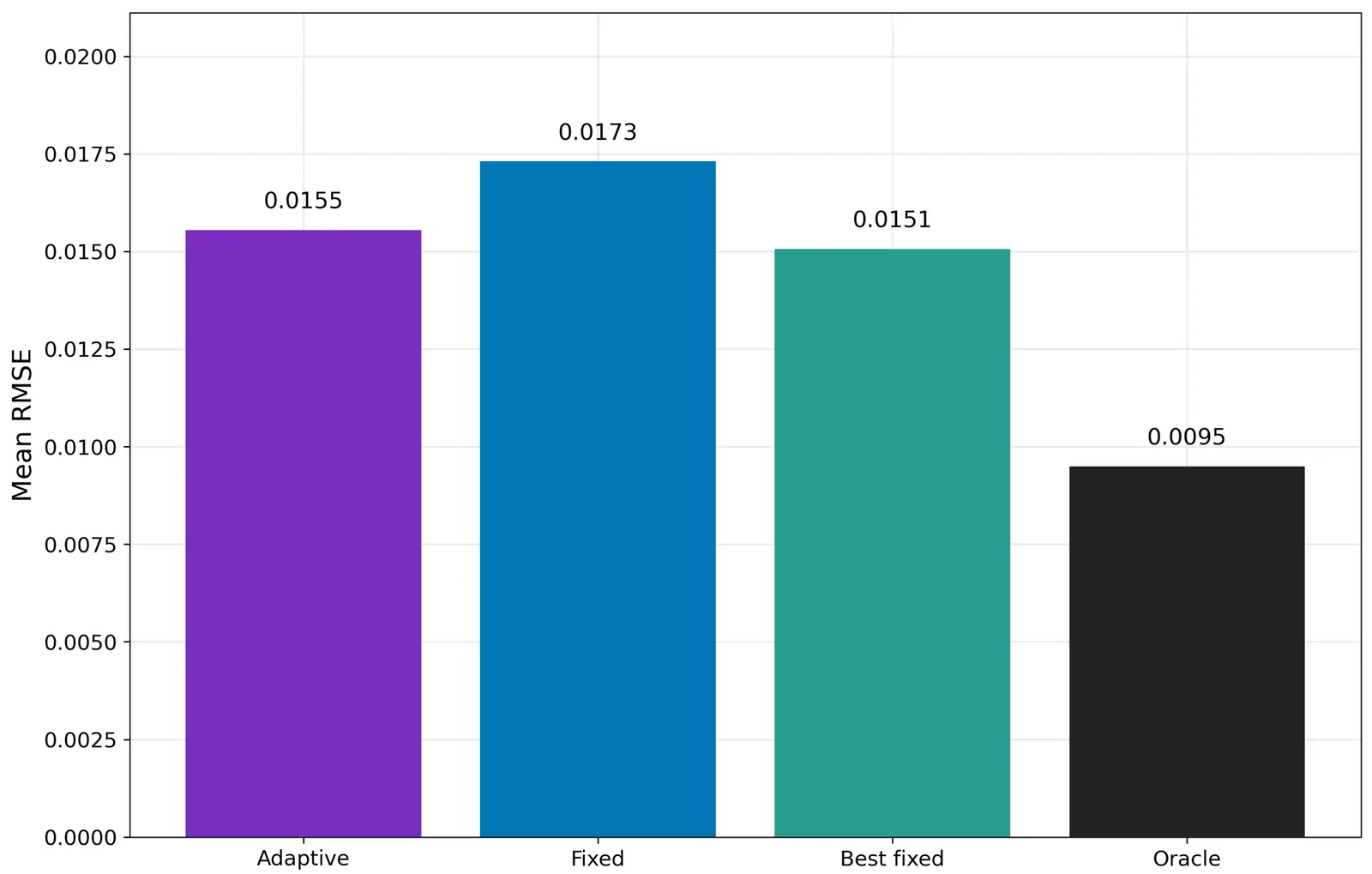
Overall RMSE comparison of the evaluated methods.

Figure 3 compares the aggregate RMSE of all evaluated methods. The proposed controller achieves a lower error than the fixed baselines, while the dynamic oracle provides the lowest RMSE.

The proposed controller has MAE 0.011391, bias $-4.85\times 10^{-5}$, error variance 0.000255, normalized RMSE 0.014327, 95th-percentile absolute error 0.030618, and maximum absolute error 0.091851. The mean switch count is 63.756 per seed-scenario trial, paired-check overhead is 0.050, and warm-up overhead is 0.0167. In the noisy-reference feedback ablation, where the controller used delayed imperfect reference measurements instead of exact synthetic-field feedback, the RMSE was 0.015731 with a mean switch count of 75.819 and oracle-probe agreement of 0.578. Further, the sliding-window UCB adaptive baseline was also evaluated to compare the proposed controller with a representative non-stationary online-selection method. This baseline achieved RMSE of 0.015474, a mean switch count of 38.350, oracle-probe agreement of 0.541, and a GHZ selection rate of 0.526 across the same 160 seed-scenario trials. The UCB baseline gives slightly lower aggregate RMSE than the proposed controller, while the proposed controller provides explicit cost-aware risk accounting, paired-check feedback refresh, and interpretable switching diagnostics. Both adaptive methods outperform the validation-selected fixed baseline.

### 4.2. Scenario-Level Results

The RMSE values at the scenario level are provided in Table 4. The performance of the proposed controller outperforms the selected fixed baseline in seven out of eight scenarios. This is most apparent in a constant low noise scenario, in which the proposed controller uses GHZ very often. This is the only scenario in which the performance is not improved by the proposed approach, as the RMSE value is 0.020060 compared with 0.019029.

A local hyperparameter sensitivity analysis was also performed to test whether the result depends strongly on a single controller setting. The analysis used five seeds, all eight scenarios, and 40 seed-scenario trials per configuration. The baseline configuration in this reduced sensitivity study achieved RMSE 0.015655 compared with 0.017424 for the validation-selected fixed baseline, corresponding to a 10.16% relative improvement. Table 5 shows that the controller remains better than the validation-selected fixed baseline across the tested local variants, although performance changes with feedback delay, paired-check period, and window size.

**Figure 4:**
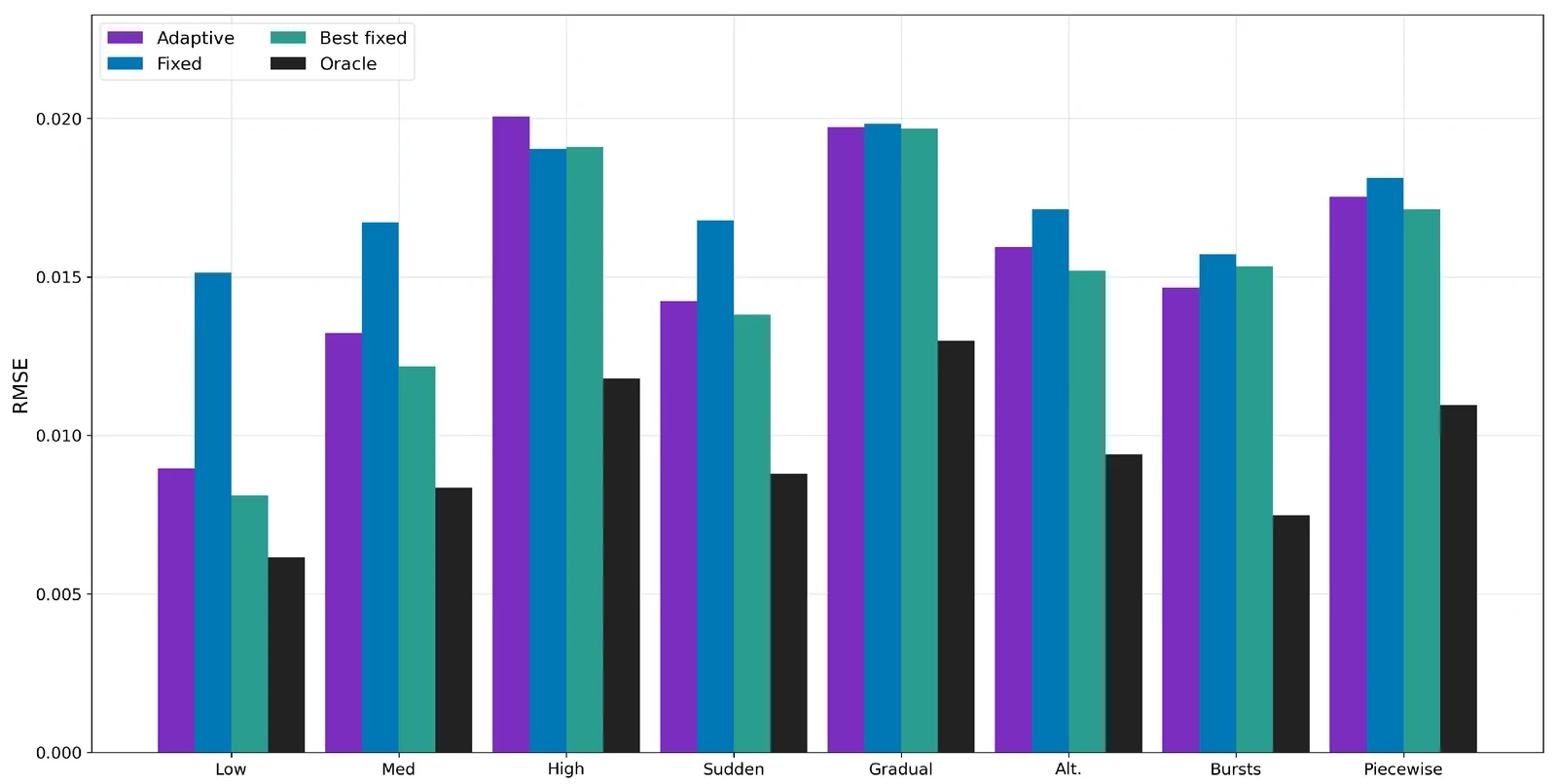
Scenario-wise RMSE comparison under different noise conditions.

Figure 4 compares the controller performance across different noise scenarios. The proposed method performs best in low- and medium-noise conditions, while a small performance drop is observed under persistent high noise.

**Figure 5:**
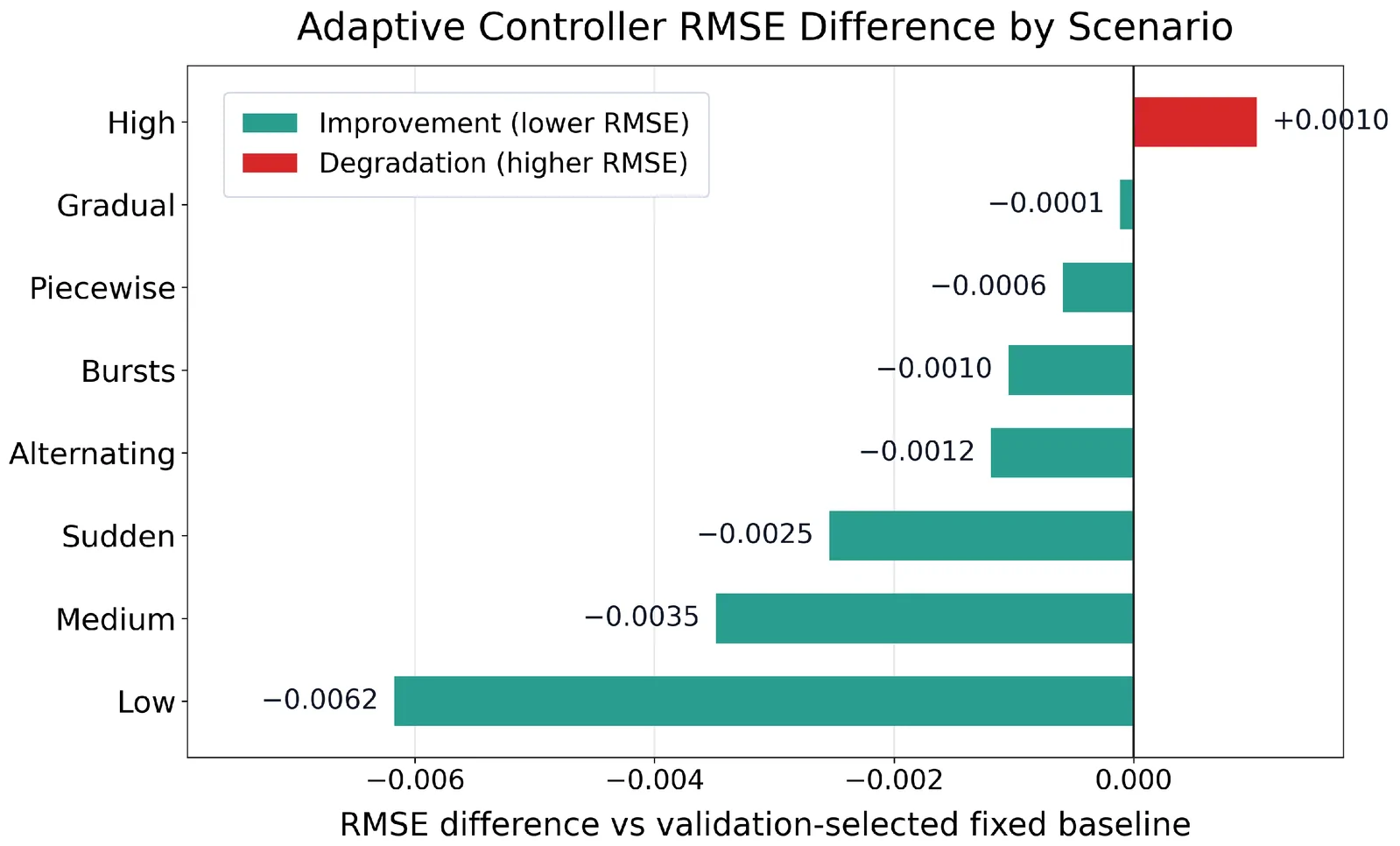
RMSE difference relative to the fixed baseline.

Figure 5 shows the RMSE difference between the proposed controller and the validation-selected fixed baseline across all scenarios. Negative values indicate that the proposed method achieves lower estimation error.

### 4.3. Probe Selection and Switching Behavior

**Figure 6:**
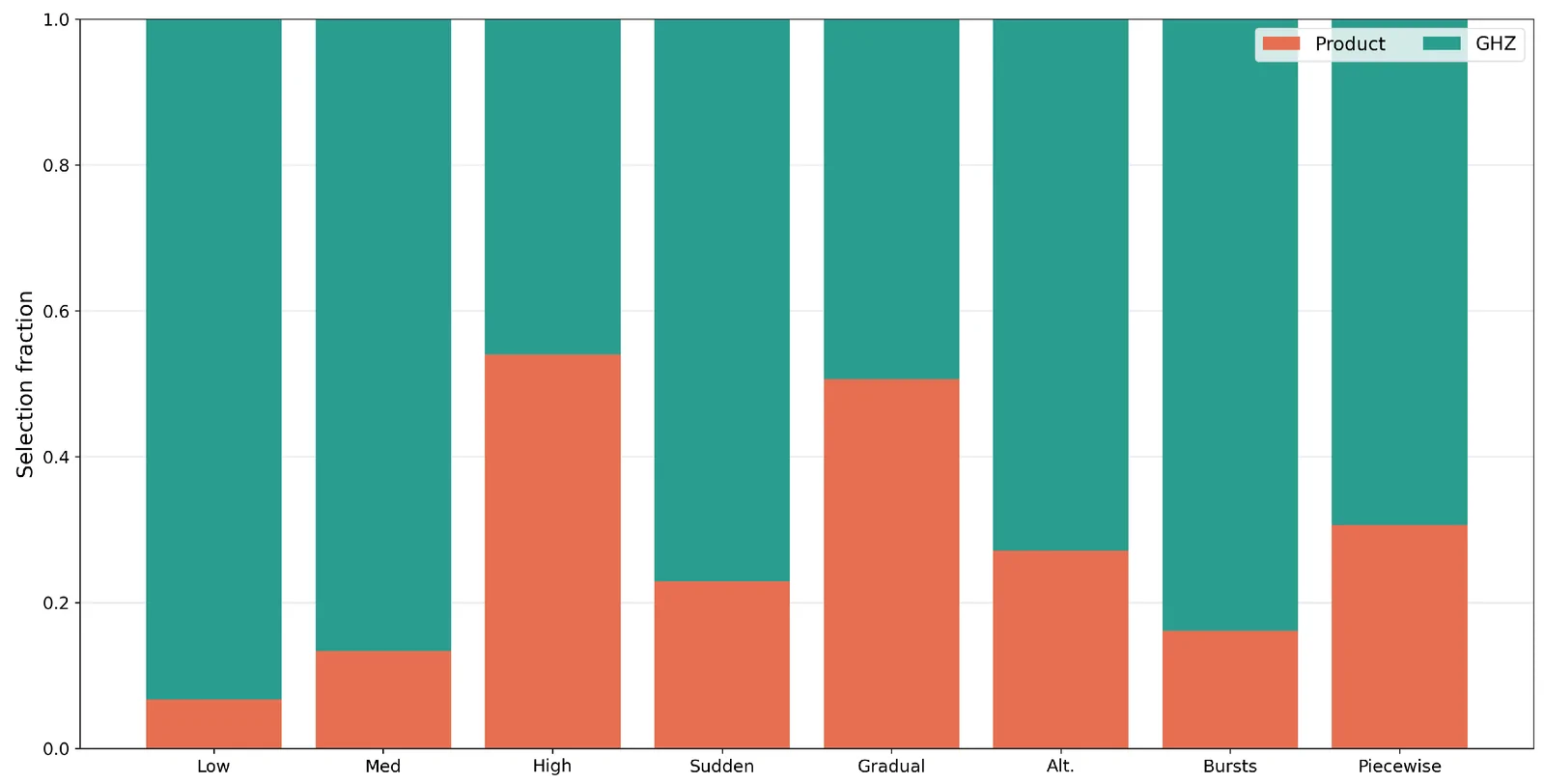
Probe usage across noise scenarios.

**Figure 7:**
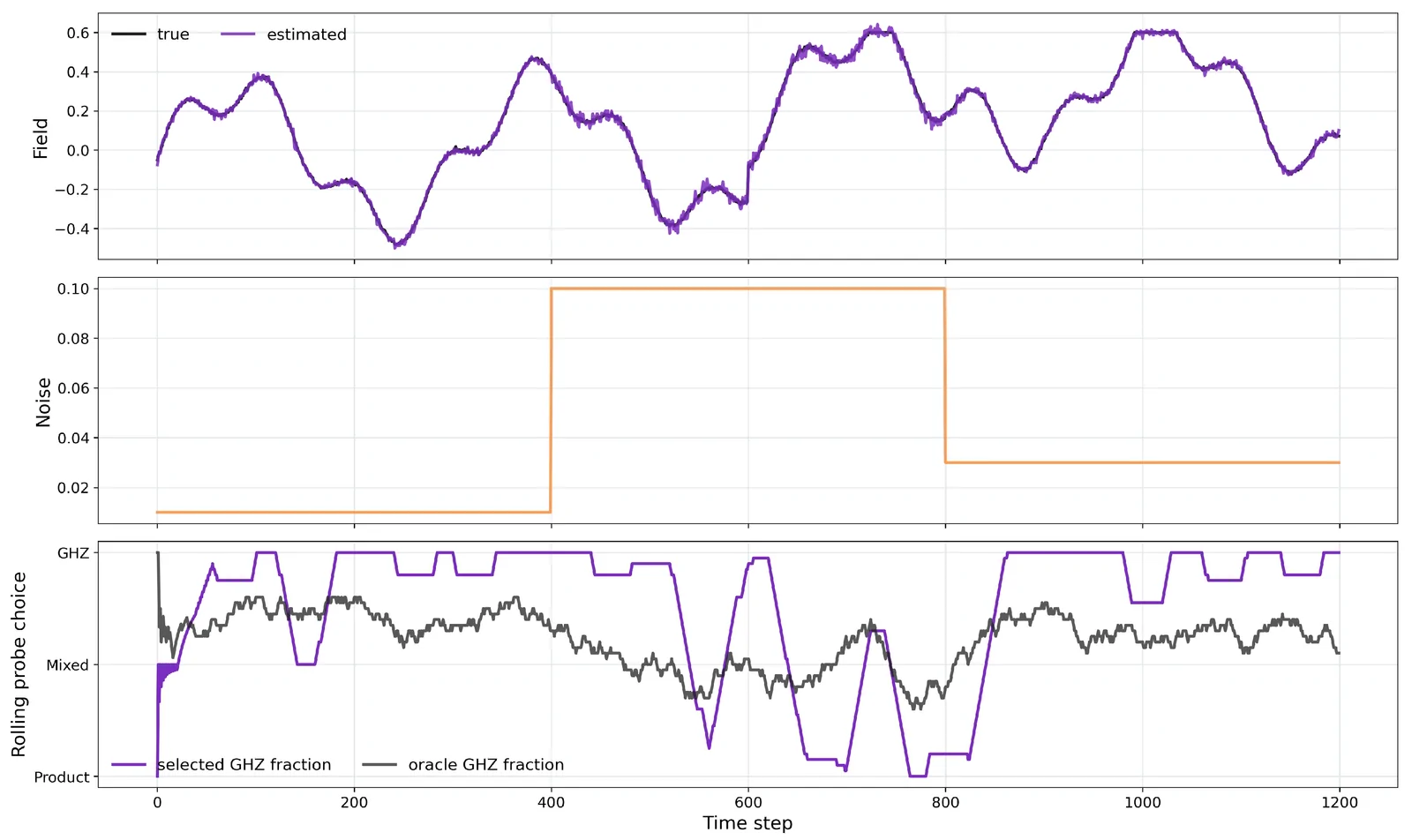
Representative trajectory under sudden noise change.The top panel compares the true field and estimated field, the middle panel shows the dephasing noise level, where the orange line denotes the noise probability, and the bottom panel shows the rolling selected and oracle GHZ fractions.

**Figure 8:**
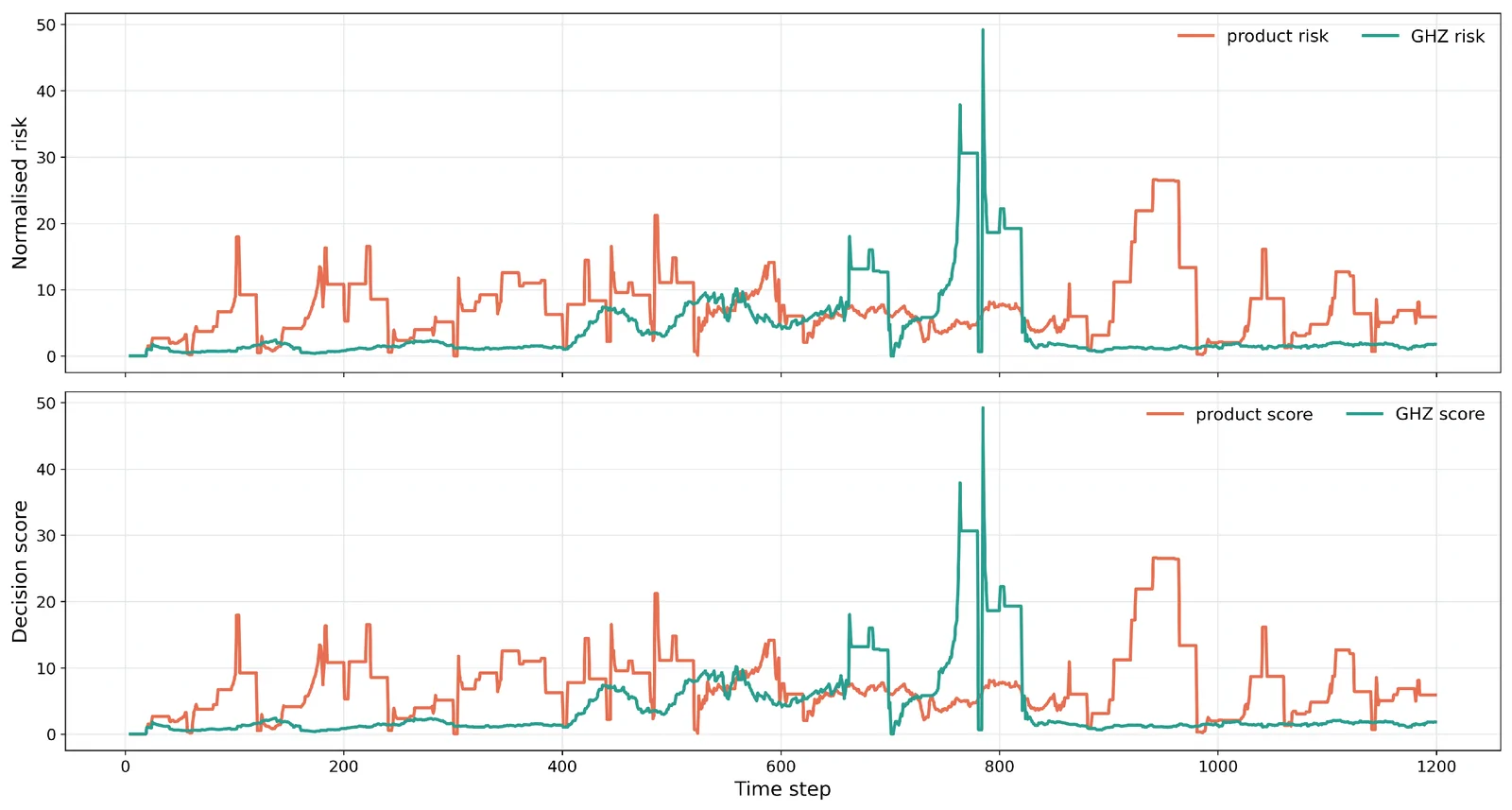
Controller risk and decision-score evolution.

**Figure 9:**
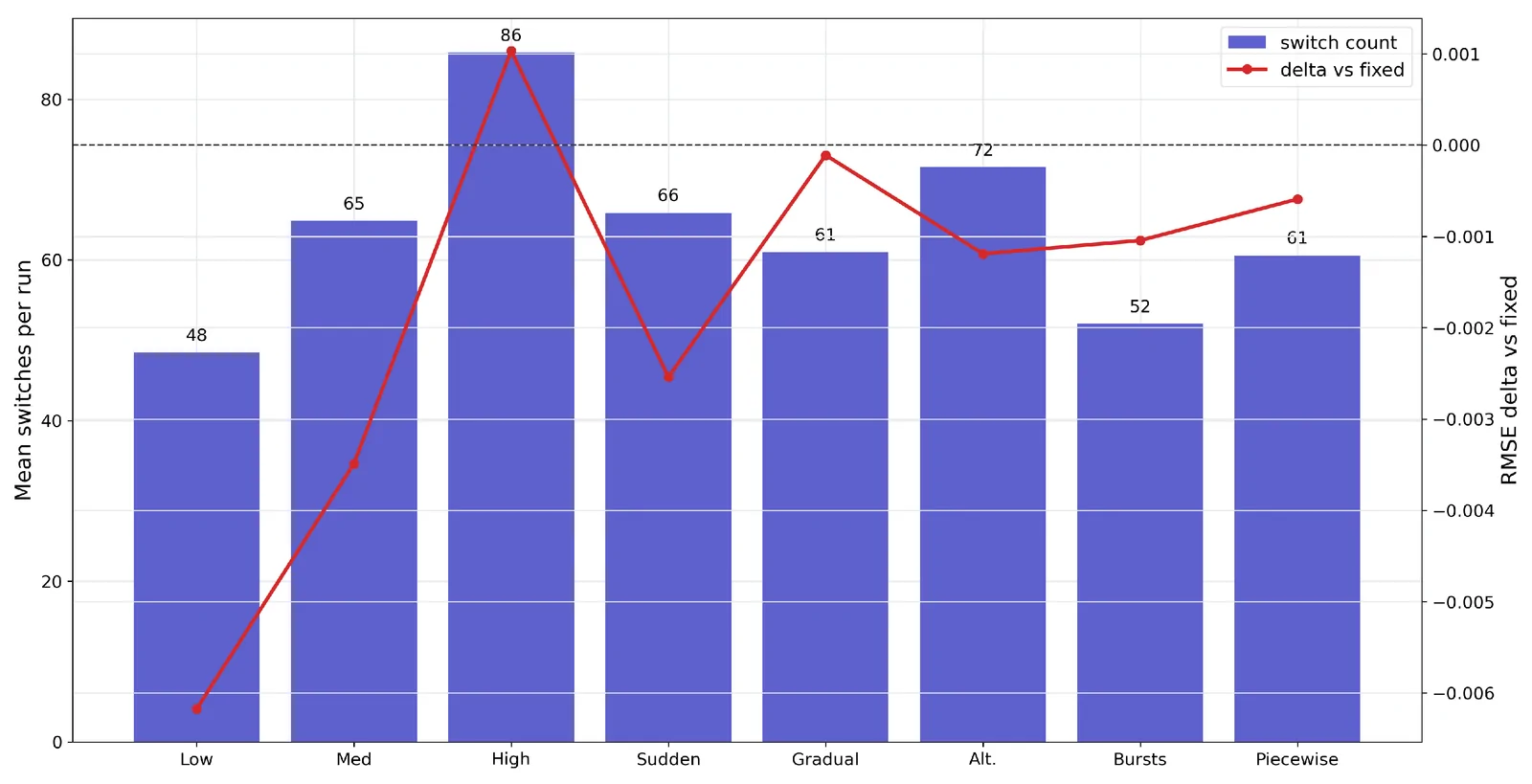
Switching trade-off across noise scenarios.Blue bars show the mean switch count per run. The solid red line shows the RMSE difference relative to the validation-selected fixed baseline, and the dotted black horizontal line marks zero RMSE difference.

Usage indicates that the adaptation was indeed achieved following the desired pattern. The GHZ usage is higher in the constant-low and constant-medium situations, whereas product usage is higher in the high-noise and gradual cases. The average GHZ selection probability for the low-noise case is 0.810, return-to-low is 0.663, and product selection diagnostic for high-noise case is 0.248 over the predefined transition intervals. The total oracle-probe correlation is 0.590, which is significant compared to chance but still far from the dynamic-oracle bound. Figure 6, Figure 7, Figure 8 and Figure 9 represent the adaptive characteristics of the developed controller. Figure 6 represents probe usage in various noise situations; note the higher GHZ usage in the low-noise regime. Figure 7 provides an example of the sudden change trajectory, including the true and estimated field, absolute error, and GHZ selection characteristics. Figure 8 displays risk estimations and decision scores over delayed feedback. Figure 9 illustrates the switching trade-off across the considered noise scenarios.

### 4.4. Statistical Tests

**Figure 10:**
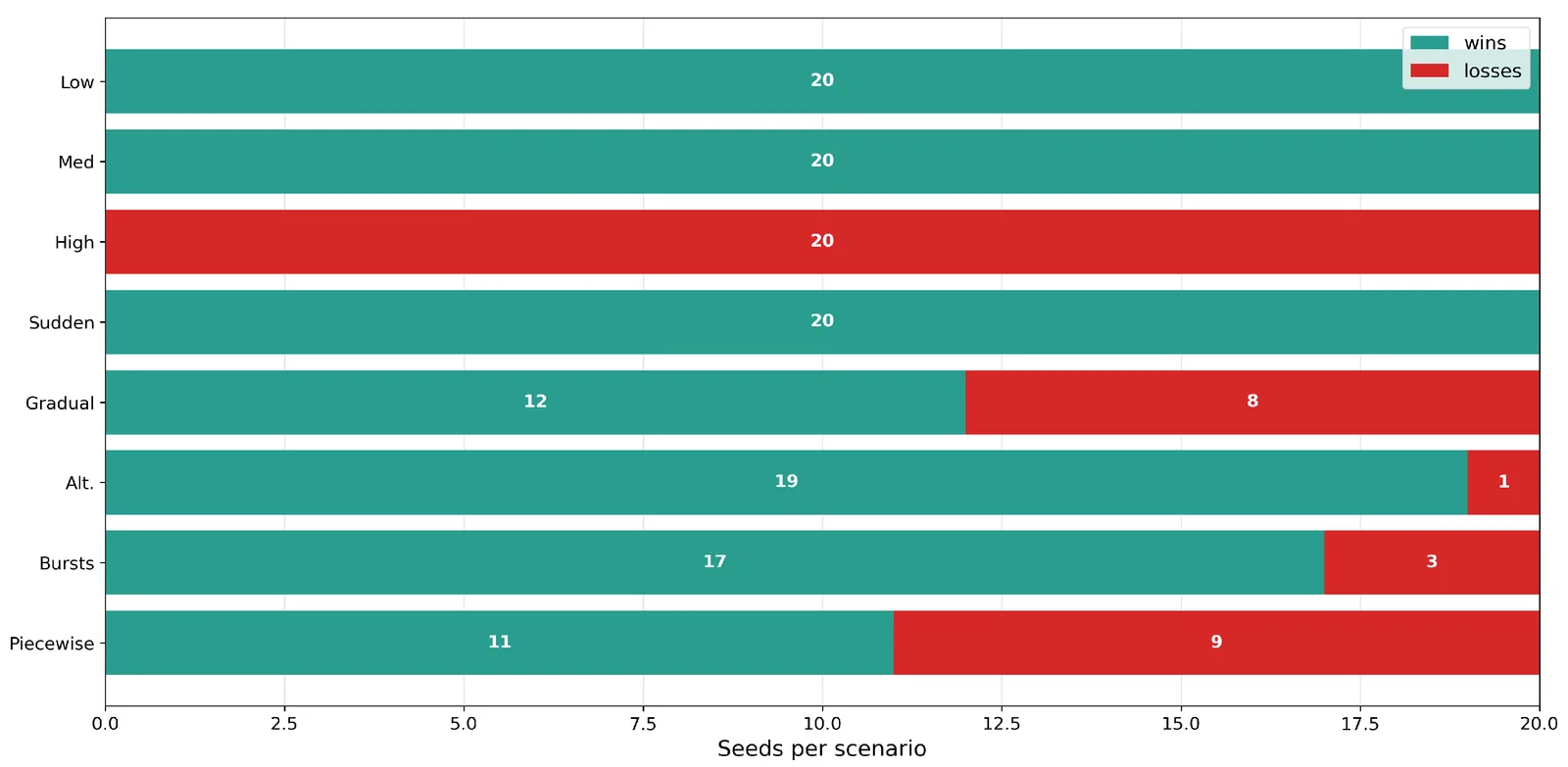
Paired win/loss comparison against fixed baselines.

Paired comparison between the new controller and the fixed one selected via validation includes 160 paired comparisons. The mean of differences between paired RMSEs equals −0.001763, the median equals −0.001487, and the 95% confidence interval is [−0.002132,−0.001404]. The new controller outperforms the other one in 119 of 160 paired seed-scenario comparisons. The paired permutation test gives p=0.000999, and the Wilcoxon signed-rank test gives $p=5.85\times 10^{-15}$. Figure 10 summarizes the paired win/loss comparison against the fixed baselines. The proposed controller wins most seed-scenario comparisons against the validation-selected fixed baseline.

## 5. Discussion

The findings provide empirical evidence for the central claim that the online probe switch is able to enhance finite-shot four-qubit magnetometry under nonstationary noise dynamics. The overall gain relative to the fixed strategy, which has been chosen based on the validation set, is statistically significant and occurs under sudden, alternating, burst, random-piecewise, constant-low, constant-medium, and gradual conditions. The dynamic-oracle difference reveals that the current controller is suboptimal but interpretable.

Improvement over the fixed baseline chosen from the validation data is in the order of 10.2% in RMSE. With noisy-reference feedback, the gain is still in the order of 9.1%. Both these improvements are both statistically significant and practical in the context of the normalized simulation model. However, they have to be understood in conjunction with the extra resources that the controller uses, including about 64 switches per trial and 5% check pairing overhead. The illustrative NV-center mapping further shows how the normalized estimation error can be translated into a physically meaningful magnetic-field scale once a specific gyromagnetic ratio and interrogation time are selected; however, this mapping is not intended as a prediction of experimentally achievable sensitivity.

The Fisher information approach provides an explanation for the switching phenomenon. GHZ has a definite low noise regime because of phase coherence through the collective effect, but product states benefit from the effect of dephasing and therefore are able to compete with GHZ states. This numerical switch at p≈0.18 suggests that the control system senses the tradeoff between sensitivity and probes.

Further, the sliding-window UCB benchmark suggests that the application of a standard online adaptive selection scheme proves effective in this environment. The overall RMSE value of the benchmark is slightly lower than the RMSE value of the controller being proposed. In terms of the claim made in this paper, this implies that the major benefit of the controller under consideration does not lie in its higher effectiveness in RMSE terms compared to all adaptive schemes but in the development of a comprehensible switching cost-sensitive model featuring checks and diagnostics of the resources utilized.

Furthermore, The constant-high case is the key scientific vulnerability. The suggested controller achieves RMSE 0.020060, while the validation-chosen fixed reference gets 0.019029. This means that it is a 5.4% worse performance and leads to one criterion failure specific for the scenario. As seen from the probe usage table, the controller still uses 46% of the selections for GHZ in the constant-high noise case. The probable cause is that the use of paired tests and estimators with finite window sizes retains the exploration and delayed evidence, which is useful in varying conditions, but not efficient in the case of persistent high noise. A deployable version should therefore include stronger high-noise persistence detection, adaptive paired-check frequency, or a confidence-based product lock that activates only after estimator-validated evidence.

Additionally, the graph-state experiment must be considered only a diagnostic ablation because the graph state and the measurements in use were not optimized for the task under the noise model; hence, the high RMSE = 0.061184. Thus, the result cannot serve as a basis for excluding the graph states. In addition, the resource aspect is equally important. The mean number of switches is 63.756, and the overhead due to the use of paired-check is 5%. Overhead is acceptable in simulations but requires calibrations for the corresponding hardware in hardware-based experiments. The preparation cost is simplified intentionally and must be adjusted to hardware values.

## 6. Limitations

The following limitations must be mentioned. Firstly, all the data is artificial and produced according to the selected density-matrix model. The major controller relies upon delayed reference feedback from the predefined simulated field; hence, this is controlled simulation, not a completely referenceless hardware experiment. However, a noisy-reference feedback test was performed to lower the effect. A practical feedback will be needed for any sensor in the form of a calibration period, reference sensor, or self-consistency signal.

Secondly, the simulations include only four qubits. Thirdly, the estimator employs the calibrated response curves and the restricted field range; the broader ranges might require phase unwrapping or Bayesian filters. Fourthly, only the local dephasing and readout flip noise are considered, whereas correlated noise, amplitude damping, leakage, crosstalk, and calibration drifts are neglected. Fifthly, the tuning of the controller has been done in the course of this research; hence, it is necessary to perform tests for other families of scenarios. Sixthly, the behavior of the system in the case of persistent high noise is still worse compared to the globally-fixed baseline.

Lastly, the presented RMSE values are normalized errors in simulations. To estimate the absolute magnetic-field sensitivity, the information about the particular platform of the sensor and its gyromagnetic ratio, interrogation time, coherence time, and measurement bandwidth is required.

## 7. Conclusions and Future Scope

This paper has presented the exploration of a cost-aware adaptive switching strategy for four-qubit quantum magnetometry in stationary and non-stationary noise scenarios. On an average over 160 trials of different seed scenarios, the proposed sliding-window control method yielded a lower RMSE of 0.015542 compared to a fixed benchmark chosen through validation (RMSE = 0.017305), resulting in an approximate gain of 10.2%. The superiority of the sliding window control was confirmed also using a paired permutation test (p=0.000999). The controller performed better than the fixed benchmark in seven out of eight examined noise scenarios, demonstrating the largest gain for low- and mixed-noise scenarios, where GHZ probes are clearly better for sensing. In turn, the noisy reference feedback experiment confirmed the effectiveness of the controller even under the substitution of perfect field feedback information with the noisy and delayed one. Also, the Fisher information analysis explained the switching behavior of the controller physically by showing higher sensitivities of GHZ probes to low dephasing, while product probes tend to be more useful with an increasing level of dephasing. Simultaneously, a smaller RMSE value of the sliding-window UCB controller suggests that the proposed approach should rather be viewed as a cost-aware and interpretable competitor of the fixed baseline than an unconditionally superior adaptive probe selector.

The example with the NV-center offers a physical interpretation of normalized error values. Given a certain gyromagnetic ratio and interrogation time, the normalized RMSE can be translated into a magnetic-field error. Nonetheless, this is just an illustration of the physical meaning of the normalized error value and does not imply the demonstration of any experimentally achieved sensitivity, since the actual performance of the system also depends on specific platform coherence, readout, bandwidth, control, and hardware noise properties.

Thus, the results of the study confirm the efficiency of adaptive probe selection for finite-shot quantum-sensing simulations in changing noise conditions. However, the current study is restricted to a normalized simulation of a four-qubit magnetometer, and the controller yields poor results under persistent high-noise scenarios in comparison with a validation-selected fixed baseline. Further research should hence focus on high-noise detection; adaptive paired-check scheduling; the large qubits system; improved graph-state measurement; hardware calibration of preparation and switching costs; and more realistic noise models such as correlated, non-Markovian, and experimentally motivated stochastic processes.

## Figures and Tables

**Table 1:** Representative classical Fisher-information proxy for product and GHZ probes under local dephasing.

Dephasing *p*	Product FI	GHZ FI	Preferred Probe
0.00	0.766	10.291	GHZ
0.05	0.484	3.372	GHZ
0.10	0.353	1.231	GHZ
0.15	0.259	0.413	GHZ
0.18	0.213	0.201	Product
0.20	0.185	0.119	Product

**Table 2:** Sliding-window probe-switching procedure with delayed reference-feedback update.

Sliding-Window Probe Switching
Initialize product and GHZ histories using alternating warm-up measurements.
At time *t*, compute ${\hat{R}}_{a,t}$ for each probe using the latest 40 delayed feedback errors.
If *t* is a paired-check step, split the shots between product and GHZ and update both histories after the feedback delay.
Otherwise, select $a_{t}=\arg\min_{a}S_{a,t}$.
Simulate field encoding, dephasing, readout error, shot sampling, and field estimation.
For the main benchmark, store delayed reference squared-error feedback computed from the synthetic field trajectory.
For the noisy-reference ablation, store delayed feedback computed as ${\left({\hat{B}}_{a,t}-B_{t}^{\mathrm{ref}}\right)}^{2}$, where $B_{t}^{\mathrm{ref}}=B_{t}+\epsilon_{t}$ and $\epsilon_{t}∼N\left(0,\;0.01^{2}\right)$.
Update the switching and resource counters.

**Table 3:** Aggregate RMSE across 160 seed-scenario trials. Lower is better.

Method	RMSE
Dynamic oracle	0.009488
Scenario-wise hindsight fixed	0.015066
Sliding-window UCB	0.015474
Proposed sliding-window controller	0.015542
Validation-selected fixed	0.017305
Fixed product	0.017330
Fixed GHZ	0.017821
Fixed graph	0.061184

**Table 4:** Scenario-level RMSE and probe usage.

Scenario	Proposed	Global Fixed	Hindsight Fixed	Dynamic Oracle	Product Use	GHZ Use
Alternating	0.015944	0.017133	0.015197	0.009411	0.271	0.729
Bursts	0.014662	0.015706	0.015338	0.007475	0.161	0.839
Constant high	0.020060	0.019029	0.019091	0.011796	0.540	0.460
Constant low	0.008962	0.015136	0.008101	0.006147	0.067	0.933
Constant medium	0.013227	0.016714	0.012181	0.008348	0.134	0.866
Gradual	0.019717	0.019827	0.019681	0.012978	0.507	0.493
Random piecewise	0.017525	0.018116	0.017137	0.010959	0.306	0.694
Sudden	0.014237	0.016777	0.013806	0.008788	0.229	0.771

**Table 5:** Local hyperparameter sensitivity analysis.

Configuration	RMSE	Global Fixed RMSE	Improvement (%)	Switch Count
Baseline W40 PC20 SW0.001 D5	0.015655	0.017424	10.16	62.15
Window W20	0.016093	0.017424	7.64	101.20
Window W60	0.016211	0.017424	6.96	44.08
Switch cost 0	0.015654	0.017424	10.16	62.15
Switch cost 0.005	0.015681	0.017424	10.01	63.00
Paired check 10	0.015955	0.017424	8.43	49.50
Paired check 40	0.015693	0.017424	9.94	67.33
Delay 0	0.015352	0.017424	11.89	45.63
Delay 10	0.016490	0.017424	5.36	71.33

## Data Availability

The data supporting the findings of this study were generated through numerical simulations. The simulation code and generated data will be made available by the corresponding author upon reasonable request.
